# The use of taxon-specific reference databases compromises metagenomic classification

**DOI:** 10.1186/s12864-020-6592-2

**Published:** 2020-02-27

**Authors:** Vanessa R. Marcelino, Edward C. Holmes, Tania C. Sorrell

**Affiliations:** 1 Marie Bashir Institute for Infectious Diseases and Biosecurity and Faculty of Medicine and Health, Sydney Medical School, Westmead Clinical School, The University of Sydney, Sydney, NSW 2006 Australia; 2 Centre for Infectious Diseases and Microbiology, Westmead Institute for Medical Research, Westmead, NSW 2145 Australia; 3 School of Life & Environmental Sciences, Charles Perkins Centre, The University of Sydney, Sydney, NSW 2006 Australia

**Keywords:** Reference database, Fungi, Microbiome, Metagenomic classifier, Misclassification, Assembly errors

## Abstract

A recent article in *BMC Genomics* describes a new bioinformatics tool, HumanMycobiomeScan, to classify fungal taxa in metagenomic samples. This tool was used to characterize the gut mycobiome of hunter-gatherers and Western populations, resulting in the identification of a range of fungal species in the vast majority of samples. In the HumanMycobiomeScan pipeline, sequence reads are mapped against a reference database containing fungal genome sequences only. We argue that using reference databases comprised of a single taxonomic group leads to an unacceptably high number of false-positives due to: (i) mapping to conserved genetic regions in reference genomes, and (ii) sequence contamination in the assembled reference genomes. To demonstrate this, we replaced the HumanMycobiomeScan’s fungal reference database with one containing genome sequences of amphibians and reptiles and re-analysed their case study. The classification pipeline recovered all species present in the reference database, revealing turtles (Geoemydidae), bull frogs (Pyxicephalidae) and snakes (Colubridae) as the most abundant herpetological taxa in the human gut. We also re-analysed their case study using a kingdom-agnostic pipeline. This revealed that while the gut of hunter-gatherers and Western subjects may be colonized by a range of microbial eukaryotes, only three fungal families were retrieved. These results highlight the pitfalls of using taxon-specific reference databases for metagenome classification, even when they are comprised of curated whole genome data. We propose that databases containing all domains of life provide the most suitable option for metagenomic species profiling, especially when targeting microbial eukaryotes.

## Background

Identifying organisms in metagenome samples remains challenging, especially when working with microbial eukaryotes. Current methods for taxonomic identification of metagenomes can be classified into two categories: assembly-based approaches (yielding metagenome-assembled genomes) and read- (or k-mer) mapping approaches. Assembly-based methods have enabled the discovery and accurate taxonomic identification of a range of bacteria and archaea (e.g. [1]), viruses (e.g. [2]) and, more recently, eukaryotes [3]. Obtaining eukaryotic metagenome-assembled genomes requires very deep sequencing, given their large genome sizes and typically low relative abundances. Mapping short sequence reads directly to a reference database is a more sensitive approach to detect rare microorganisms and is usually preferred for taxonomic profiling and comparative studies (reviewed in [4]).

A recent article by Soverini and colleagues [5] describes HumanMycobiomeScan – a new bioinformatics tool to identify fungi in metagenome samples based on mapping sequence reads to a reference database of fungal genomes. The authors applied the pipeline to characterize the gut mycobiome of human populations on different diets (hunter-gatherers and Western populations), resulting in the identification of a range of fungal taxa in 37 of 38 samples analysed. Briefly, the pipeline consists of aligning short sequence reads to a reference database with Bowtie2 [6], filtering human and bacterial reads with BMTagger [7], and re-mapping the high-quality reads to a fungal reference database to obtain taxonomic assignments and abundance estimates. The default reference database contains the 66 complete fungal genomes available in NCBI in 2018. Similar taxon-specific reference databases have been implemented in other metagenome classification tools. The FindFungi pipeline for example [8] is based on a Kraken [9] reference database composed of 949 fungal genomes.

The choice of reference database directly impacts diversity and composition inferences from metagenome data [10]. We argue that using taxon-specific reference databases in assembly-free metagenome classification pipelines, such as implemented in HumanMycobiomeScan, leads to an unacceptably high number of misclassifications. Here we demonstrate and discuss the pitfalls of using databases constrained to specific taxon groups, and propose that kingdom-agnostic approaches provide a more accurate metagenome classification.

## Analyses and results

We re-analysed the 38 metagenomes used as a case study in the HumanMycobiomeScan publication [5]. These were obtained from stool samples of Hadza hunter-gatherers from Tanzania (*n* = 27) and Western subjects from Italy (*n* = 11) [11].

To demonstrate the potential pitfalls of using a taxon-specific reference database, we replaced the fungal database in the HumanMycobiomeScan pipeline with a database containing organisms that do not normally occur in the human gut (Additional file 1). Specifically, we built a reference database containing genomes of 18 species of amphibians (frogs and salamanders) and reptiles (snakes, lizards, geckos, turtles and crocodiles). Default values were used for all other HumanMycobiomeScan parameters (see Additional file 2 for details).

**Fig. 1:**
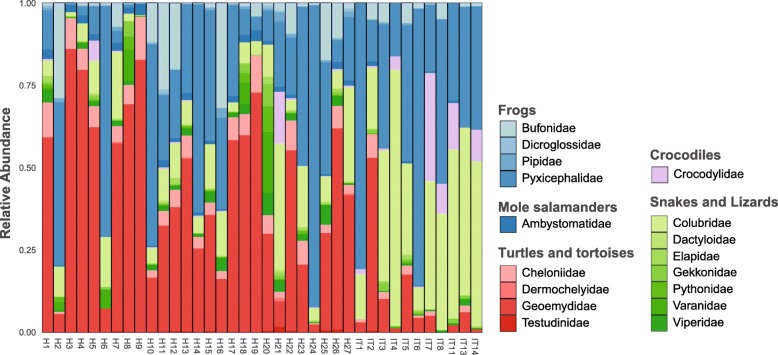
Herpetofaunal diversity identified in human stool samples of Hadza hunter-gatherers and Western populations using the HumanMycobiomeScan pipeline with a reference database containing genomes of amphibians and reptiles, demonstrating the pitfalls of using taxon-specific reference databases

HumanMycobiomeScan identified a range of amphibians and reptiles in all the samples analysed, with turtles (Geoemydidae), bull frogs (Pyxicephalidae) and snakes (Colubridae) suggested to be the most abundant herpetological taxa in the human gut (Fig. 1). Remarkably, all species in the reference database were identified in 12 or more samples. The frog species *Pyxicephalus adspersus* and the snake species *Pantherophis guttatus* were identified in all samples tested. These results are comparable to the findings of Soverini and colleagues, where 65 fungal species were retrieved when using a database containing 66 fungal genomes [5].

**Fig. 2:**
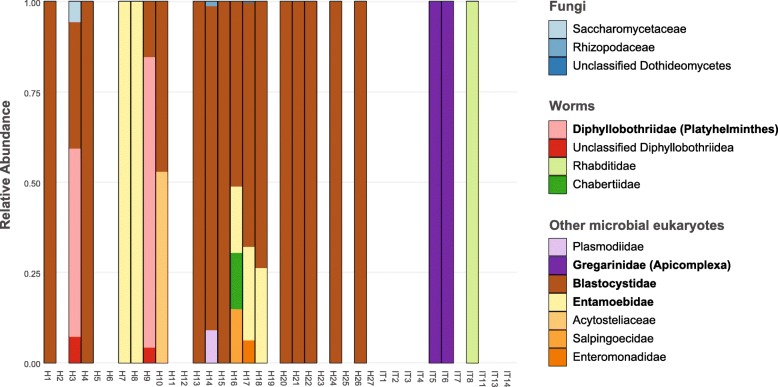
Microbial eukaryotes reported in the gut microbiome of hunter-gatherers and Western subjects using the CCMetagen pipeline and the NCBI nucleotide collection as reference. The graphs show the diversity and relative abundance of microbial eukaryotes only, with other organisms removed. Microbial eukaryotes were detected in 21 of 38 samples, although some of these are likely false-positives. The most abundant families observed across all samples are highlighted in bold

We also analysed the case study using the entire NCBI nucleotide collection (NCBI nt) as a reference and the kingdom-agnostic CCMetagen metagenomic pipeline [12]. Instead of making taxonomic inferences based on individual sequence reads as is done with Bowtie, the CCMetagen pipeline uses KMA [13], which takes advantage of the information from all high-scoring read mappings to determine which reference sequence is the most likely match. The analysis revealed a range of bacteria, archaea, viruses (bacteriophage) and eukaryotes (Additional file 3). Among the eukaryotes identified, we observed obvious misclassifications, including insects, mollusks and plants, which may reflect misidentifications in the NCBI nt reference database [14]. To focus on microbial eukaryotes, we filtered out taxonomic assignments belonging to the Arthropoda, Mollusca, Streptophyta, Chordata (including humans), Bacteria, Archaea and viruses (Additional file 2). From these data we detected microbial eukaryotes in 21 subjects. Fungi were detected in only three hunter-gatherers (Fig. 2), comprising Saccharomycetes, Mucoromycetes (Rhizopodaceae) and Dothideomycetes. Species-level identifications could not be retrieved for any of these fungal taxa. Saccharomycetes was the most abundant fungal class with 36 sequence reads. The remaining two fungal taxa were present at extremely low abundance, with only two sequence reads assigned to Mucoromycetes and a single read assigned to Dothideomycetes, making it possible that they represent misclassifications. We additionally retrieved a range of microbial eukaryotes known to colonize the human gut, including the protozoan *Blastocystis*, parasitic worms (Platyhelminthes and Nematodes), Amoebae and Apicomplexans (Fig. 2). We note, however, that it is likely that some of these also represent false-positives due to contamination in reference databases.

We further investigated the causes of misclassifications by attempting to identify the origins of sequences classified as fungi by HumanMycobiomeScan. Specifically, we analyzed the 101 sequences from sample H1 belonging to fungi (25 fungal genera) according to HumanMycobiomeScan. Using BLAST [15], we found that 85% of these putative fungal sequences were rRNA genes (Additional files 2 and 4) that are highly conserved across domains of life. Part of the sequences aligned to unspecified genomic regions (11%), and only 4% to protein-coding genes. Of the 101 putative fungal sequences, only one was also classified by CCMetagen: this sequence was identified as *Blastocysts* sp. rRNA by both CCMetagen and BLAST analyses.

## Discussion

Here, we show that using taxon-specific reference databases in assembly-free taxonomic profiling, such as in the HumanMycobiomeScan, can lead to identification of organisms that are demonstrably not present in a metagenome. Although the HumanMycobiomeScan pipeline includes a quality-control step to remove bacterial and human sequences, this is performed after the initial taxonomic assignment and does not prevent misclassifications. The most likely causes of this high number of false-positives are conserved genetic regions in reference genomes as well as ‘contamination’ of genome assemblies.

Genes involved in fundamental biological processes change little over evolutionary time and across taxa, and homologues of some of the highly conserved genes, such as those that encode rRNA, are found in all eukaryotes. In assembly-free approaches sequence reads are aligned to the closest (usually homologous) reference sequence in the database, which can be a conserved gene from a distantly-related taxon. It is possible that some of the fungi identified by Soverini and colleagues are misclassifications of other abundant microbial eukaryotes in those samples, such as the protozoan *Blastocystis*. This misclassification problem is reduced when using reference databases containing a broad range of organisms, such as the NCBI nucleotide collection. Another option to mitigate this issue is to use tools that group all sequence reads that map to the same reference sequence, forming ‘pseudo-assemblies’ that may span beyond conserved genetic regions [12, 13, 16].

Reference genome sequences can also be ‘contaminated’ with bacterial, viral and human DNA, which are commonly sequenced alongside the organism of interest in high-throughput sequencing assays. These contaminant sequences can then be inadvertently assembled with the genome of the target organism. For example, over 10% of NCBI’s genome assemblies of non-primate species are contaminated with human DNA [17]. Some of these contaminants contain open reading frames, with a recent study showing that over 3000 spurious proteins originated from human contaminant sequences have been assigned to bacterial, archaeal and non-human eukaryotic organisms and deposited as such in public databases [18]. Eukaryotic genome assemblies, even those from well-studied organisms, are also commonly contaminated with bacterial DNA: over a hundred bacterial contigs were identified in the genomes of the domestic cow *Bos taurus* and the model alga *Chlamydomonas reinhardtii* [19]. Contaminating sequences in assembled genomes often derive from highly abundant genetic elements (e.g. short interspersed nuclear elements and repeats in the human genome) and common contaminants of laboratory reagents, both of which are also likely to be present in metagenome data [17, 18, 20]. We therefore expect that part of the false-positives derive from human and bacterial sequence reads that align to contaminants in reference databases. Contamination and misidentifications compromise a range of reference databases (including NCBI’s nt database), and in so doing compromise the results of all metagenome classification tools, including BLAST and CCMetagen. This problem is exacerbated when using reference databases composed of assembled genomes of a single taxonomic group, as a portion of the metagenome sequence reads have no option but to align to the contaminant reference sequence

This serves as a cautionary tale highlighting the pitfalls of using taxon-specific reference databases in assembly-free metagenome classification pipelines. We show that using a reference database restricted to fungal genomes leads to unrealistic taxonomic classifications, and that reptiles and amphibians can also be incorrectly identified in the human gut if a similarly restricted herpetological database is used. Using the NCBI nucleotide collection as reference and our kingdom-agnostic pipeline, we detected a range of microbial eukaryotes – although only three putative fungal taxa – in the metagenomes of Hadza hunter-gatherers and Western individuals. We therefore recommend using databases containing all domains of life for metagenomic species profiling, even if the focus is on particular taxonomic groups.

## Supplementary information

**Additional file 1.** Genomes of amphibians and reptiles used to construct the herpetofaunal reference database.

**Additional file 2.** Supplementary Materials and Methods.

**Additional file 3.** Taxonomic profiling of metagenome samples from hunter-gatherers and Western populations using the CCMetagen pipeline.

**Additional file 4.** BLAST results of putative fungal sequences identified with HumanMycobiomeScan in sample H1.

## Data Availability

CCMetagen source code is freely available from https://github.com/vrmarcelino/CCMetagen. The indexed reference database used with KMA and CCMetagen (ncbi_nt_no_env_11jun2019) can be downloaded from 10.25910/5cc7cd40fca8e and from its mirror at http://www.cbs.dtu.dk/public/CGE/databases/CCMetagen/.

## Abbreviations

NCBI: National center for biotechnology information
