# Supplementary material for: The use of taxon-specific reference databases compromises metagenomic classification
Source: BMC Genomics. 2020 Feb 27;21:184. doi: 10.1186/s12864-020-6592-2 (PMC7045516; doi:10.1186/s12864-020-6592-2)
Supplement: Supplementary file 2 — Additional file 2. Supplementary Materials and Methods. [file 12864_2020_6592_MOESM2_ESM.pdf]

Additional file 2:
Supplementary Materials and Methods

The use of taxon-specific reference databases compromise metagenomic classification

Vanessa R. Marcelino, Edward C. Holmes and Tania C. Sorrell

HumanMycobiomeScan analysis

Genome sequences of amphibians and reptiles (listed in Additional file 1) were downloaded from NCBI, and ‘lcl|’ was added in front of each sequence header to conform with the HumanMycobiomeScan pipeline. Bowtie v.2.3.3.1 [1] was used to construct a reference database.

To run the HumanMycobiomeScan pipeline (including when using the default reference database) it was necessary to modify the bmfilter.sh file and add the option ‘-p false’ in the line: ‘srprismopts="-b 100000000 -n 5 -R 0 -r 1 -M 7168 -p false’ as noted in the BMTagger website (https://bioconda.github.io/recipes/bmtagger/README.html). The BMTagger website notes that ‘you may find it necessary’ to perform these edits, depending on the software version and the specific operating system.

We found that the R step (where the taxonomic profiling statistics are carried out) was excessively slow. To make the analyses more efficient, the hms.sh script was edited to clean-up the intermediate file ‘final.genes.txt’ and ‘HMS_taxonomy.txt’ (which feed into HMS.R), so that they contain information from positive matches only (i.e. sequence headers without a match were removed). Specifically, after line 95 (‘cp $HMS_PATH/HMS/var/Normalising_table.txt $OUTPUT_DIR’), the following commands were added:

```
cd $OUTPUT_DIR/
awk '$3 > 0' final.genes.txt > final.genes.filtered.txt
awk '{print $1}' final.genes.filtered.txt > gene.names.txt
sed 's/lcl|//' gene.names.txt > gene.names.clean.txt
grep -Ff gene.names.clean.txt HMS_taxonomy.txt > HMS_taxonomy.clean.txt
cd results/
```

Finally, in the R script HMS.R, line #9 (genes=genes[1:(length(rownames(genes))-1),]) was commented (deactivated) to retain the last line of the input file in the analysis. This edit was needed given that this line was already removed in the previous step with awk.

None of these edits affect the results or the accuracy of HumanMycobiomeScan.

CCMetagen analysis

To analyse the samples in a kingdom-agnostic manner, the NCBI nucleotide collection was used as reference. Taxa descending from environmental eukaryotes (taxid 61964), environmental prokaryotes (taxid 48479), unclassified sequences (taxid 12908) and artificial sequences (taxid 28384) were removed from the database. The database was indexed with KMA v1.2.6 [2] using the options -NI -Sparse TG. The indexed database can be downloaded from http://dx.doi.org/10.25910/5cc7cd40fca8e and from its mirror at http://www.cbs.dtu.dk/public/CGE/databases/CCMetagen/ (file named ncbi_nt_no_env_11jun2019.zip)

KMA v1.2.6 [2] was run with the options:

```
kma -ipe <read_pair_1>1 <read_pair_2> -o <output> -t_db <ref_db_file_path> -t 4 -1t1 -mem_mode -and -apm f
```

And CCMetagen v.1.1.3 [3] was subsequently run with default options:

```
CCMetagen.py -i <input_file_path.res> -o <output>
```

To produce the taxonomic profile containing all biological kingdoms (Additional file 3), we used CCMetagen_merge:

```
CCMetagen_merge.py -i <input_folder> -o <output>
```

To filter out contaminants and human sequences, and to produce an abundance table at the family level, the ‘keep_or_remove’ feature of CCMetagen was used:

```
CCMetagen_merge.py -i <input_folder> -t Family -kr r -l Phylum -tlist Arthropoda,Chordata,Mollusca,Streptophyta -o <output>
```

Graphs were produce din R using PhyloSeq [4] and ggplot2 [5].

Inspection of putative fungal sequences

To better understand the causes of misclassifications in HumanMycobiomeScan, we further analyzed one sample (H1 - SRR1927149).

HumanMycobiomeScan analyses were performed using the default Fungi_LITE reference database. We edited the HMS pipeline to not erase intermediate files, thereby allowing us to extract the sequences identified as fungi. Specifically, we commented the following line in the HMS.sh file:

```
#rm -rf $OUTPUT_DIR/*
```

The 101 putative fungal sequences were converted to fasta using SAMtools [6]:

```
samtools bam2fq SRR1927149_H1-final.sorted.bam | seqtk seq -A - > fungi_sorted.fa
```

Blast alignment was performed using the NCBI nucleotide collection, minimum e-value of 1e-10, and a maximum number of sequence targets of 1:

```
blastn -query fungi_sorted.fa -db $db_nt -out $out_fp/H1_blastn -evalue 1e-10 -
max_target_seqs 1 -num_threads $th -outfmt "6 qseqid qlen sseqid stitle pident length
evalue sskingdoms staxids"
```

The 101 sequences were also compared with the output of CCMetagen. We filtered out the output of KMA (.frag file) to contain only sequences identified by CCMetagen (including all prokaryotes and eukaryotes, *i.e.* before the CCMetagen_merge.py step), and cross-checked with the sequences identified by HumanMycobiomeScan.

References

1. Langmead B, Salzberg SL: Fast gapped-read alignment with Bowtie 2. Nat Methods. 2012; 9:357-359.
2. Clausen P, Aarestrup FM, Lund O: Rapid and precise alignment of raw reads against redundant databases with KMA. BMC Bioinformatics. 2018; 19:307.
3. Marcelino VR, Clausen PT, Buchman J, Wille M, Iredell JR, Meyer W, et al: CCMetagen: comprehensive and accurate identification of eukaryotes and prokaryotes in metagenomic data. bioRxiv. 2019; doi:10.1101/641332
4. McMurdie PJ, Holmes S: phyloseq: an R package for reproducible interactive analysis and graphics of microbiome census data. PLoS One. 2013; 8:e61217.
5. Wickham H: *ggplot2: Elegant Graphics for Data Analysis.* Springer-Verlag New York; 2009.
6. Li H, Handsaker B, Wysoker A, Fennell T, Ruan J, Homer N, et al: The Sequence Alignment/Map format and SAMtools. Bioinformatics. 2009; 25:2078-2079.
